# Sex differences in cardiac mitochondrial respiration and reactive oxygen species production may predispose *Scn1a*^−/+^ mice to cardiac arrhythmias and Sudden Unexpected Death in Epilepsy

**DOI:** 10.1016/j.jmccpl.2024.100090

**Published:** 2024-08-22

**Authors:** Jessa L. Aldridge, Emily Davis Alexander, Allison A. Franklin, Elizabeth Harrington, Farah Al-Ghzawi, Chad R. Frasier

**Affiliations:** East Tennessee State University, Quillen College of Medicine, Department of Biomedical Sciences, Johnson City, TN, United States of America

**Keywords:** Dravet, SUDEP, Hypoxia, Glutathione, Arrhythmia, Sex differences

## Abstract

Dravet Syndrome (DS) is a pediatric-onset epilepsy with an elevated risk of Sudden Unexpected Death in Epilepsy (SUDEP). Most individuals with DS possess mutations in the voltage-gated sodium channel gene *Scn1a*, expressed in both the brain and heart. Previously, mutations in *Scn1a* have been linked to arrhythmia. We used a *Scn1a*^−/+^ DS mouse model to investigate changes to cardiac mitochondrial function that may underlie arrhythmias and SUDEP. We detected significant alterations in mitochondrial bioenergetics that were sex-specific. Mitochondria from male *Scn1a*^−/+^ hearts had deficits in maximal (*p* = 0.02) and Complex II-linked respiration (*p* = 0.03). Male *Scn1a*^−/+^ mice were also more susceptible to cardiac arrhythmias under increased workload. When isolated cardiomyocytes were subjected to diamide, cardiomyocytes from male *Scn1a*^−/+^ hearts were less resistant to thiol oxidation. They had decreased survivability compared to *Scn1a*^+/+^ (*p* = 0.02) despite no whole-heart differences. Lastly, there were no changes in mitochondrial ROS production between DS and wild-type mitochondria at basal conditions, but *Scn1a*^−/+^ mitochondria accumulated more ROS during hypoxia/reperfusion. This study determines novel sex-linked differences in mitochondrial and antioxidant function in *Scn1a*-linked DS. Importantly, we found that male *Scn1a*^−/+^ mice are more susceptible to cardiac arrhythmias than female *Scn1a*^−/+^ mice. When developing new therapeutics to address SUDEP risk in DS, sex should be considered.

## 1. Introduction

Dravet syndrome (DS) is a severe pediatric-onset epileptic encephalopathy. Characteristics of DS include severe, pharmacoresistent seizures, alongside neuropsychiatric comorbidities, such as delayed psychomotor and cognitive development [1–3]. Individuals with DS are at especially high risk of premature mortality (≤17 %) due to Sudden Unexpected Death in Epilepsy (SUDEP), one of the most common causes of death in persons with epilepsy [1–3]. While the exact mechanisms underlying SUDEP remain to be elucidated, cardiac, pulmonary, and neurological factors have all been implicated in its pathogenesis. Common contributing factors to SUDEP are believed to include cardiac ventricular arrhythmias, pulmonary apnea or edema, autonomic dysfunction, and postictal suppression of cerebral and brainstem structures [4–11]. DS is primarily caused by loss of function mutations in genes encoding subunits of voltage-gated sodium channels (VGSCs). Approximately 85 % of individuals with DS possess heterozygous loss-of-function mutations in the gene *SCN1A*, which encodes an α subunit of the VSGC type-1 (Na_V_1.1) [12]. The α subunit contains the voltage sensor and the ion-conducting pore of the VGSC channel. The *SCN1A* mutations seen in DS result in haploinsufficiency and consequentially, the loss of sufficient Na_V_1.1 function [13–16].

Attenuation of Na_V_1.1 current may be particularly detrimental to cardiac electrophysiology, as Na_V_1.1 is a fundamental membrane protein in cardiac tissues, involved in the upstroke and propagation of the cardiac action potential [14,16]. Previous research in the *Scn1a*^−/+^ mouse model of DS shows *Scn1a*^−/+^ mice are vulnerable to developing cardiac arrhythmias. In vivo electrocardiogram recordings demonstrated that compared to age-matched wild-type animals, *Scn1a*^−/+^ mice possess a long QT phenotype and experience more episodes of ventricular arrhythmias, including potentially fatal dysrhythmias such as ventricular fibrillation [17]. In some cases, these extreme disturbances to cardiac rhythm were observed immediately preceding death. In addition to the whole heart, altered electrophysiological properties have been recorded at the cardiomyocyte level in *Scn1a*^−/+^ mouse models. Ventricular cardiomyocytes from *Scn1a*^−/+^ mice are hyperexcitable, possess elevated levels of arrhythmogenic early afterdepolarizations, and have increased transient and persistent Na^+^ current density (I_Na_) [17–19]. Likewise, some of these electrical disturbances have also been observed in human patients with DS. Our previous work with induced pluripotent stem cell-derived cardiac myocytes from individuals with *SCN1A*-linked DS showed these cells possessed abnormal contractility and increases in transient current (I_Na_) [18]_._ Furthermore, we have demonstrated that an imbalance in sodium currents may underlie arrhythmias induced under increased workload in a model of SCN8A epilepsy [20]. However, the exact molecular mechanisms underlying the vulnerability to arrhythmias in *SCN1A-*linked DS have yet to be determined.

One cellular component linked to long-term homeostasis in cardiomyocytes is mitochondria (for review see [21]) and mitochondrial diseases have been linked to cardiac arrhythmia [22–24]. Due to the heart’s limited anaerobic capacity, it relies critically upon mitochondria to synthesize ATP. It has been estimated that under physiological conditions, at least 95 % of ATP generated in the heart is derived from the mitochondrial [25]. The vast majority is used to fuel the activity of ATP-dependent enzymes involved in cardiac contraction, particularly the sarcoplasmic reticulum Ca^2+^ ATPase [25]. Mitochondria also serve as cellular ion buffers, due to the presence of Na^+^ and Ca^2+^ transporters. For instance, Ca^2+^ has been implicated in matching ATP supply with demand in the heart [26]. As a result, in DS, increases in I_Na_ may negatively affect mitochondrial ion homeostasis as changes to Na^+^/Ca^2+^ exchange have been shown to occur. Targeting mitochondrial calcium uptake with teriflunomide has also been shown as potentially protective in the brain of DS [27], however in the heart it may have negative effects on cardiac outcomes [28]. Finally, mitochondria contribute significantly to ROS production within cells. Our previous work in the heart suggests that mitochondrial-derived ROS plays a role in pathological conditions [29,30] and imbalances between ROS production and scavenging negatively affect cardiac energetics and can lead to arrhythmia. Furthermore, our previous work demonstrates that ROS production is increased in *Scn1b-*linked DS [31].

The goal of this study was to determine if altered cardiac mitochondrial energetics and ROS production exist in the *Scn1a*^−/+^ mouse model of DS, predisposing them to cardiac arrhythmias and SUDEP events. Specifically, we examined whether *Scn1a*^+/−^ mice exhibit differences in mitochondrial respiratory pathways, imbalances in ROS production and scavenging, and overall are more susceptible to cardiac arrhythmia development. Our results indicate male *Scn1a*^−/+^ mice possess unique deficits in mitochondrial bioenergetics and ROS buffering, with male *Scn1a*^−/+^ mice predominantly affected. These observations could render males with *Scn1a*-linked DS uniquely vulnerable to adverse cardiac events or SUDEP.

## 2. Materials and methods

### 2.1. Mice

All animal procedures were approved by the Institutional Animal Care and Usage Committee of East Tennessee State University and in conformity with the NIH Guide for the Care and Use of Laboratory Animals. Animals underwent euthanasia via CO_2_. 129-*Scn1a*^*tm1Kea*^ mice (Jackson Laboratory #037107-JAX) were maintained by breeding 129-*Scn1a*^−/+^ mice to 129S6/SvEvTac mice (Taconic). Experimental mice were generated as F1 hybrids by breeding 129-*Scn1a*^−/+^ mice to C57/Bl6J mice.

### 2.2. Mitochondrial isolation, respiration, and reactive oxygen species detection

To assess respiratory chain activity, mitochondria were isolated from P20 – P25 ventricular samples *Scn1a*^−/+^ and *Scn1a*^+/+^, and mitochondrial oxygen and H_2_O_2_ flux were concurrently measured using an O2k-FluoRespirometer (Oroboros) [31]. Mitochondrial protein content was assayed with a Bradford assay and equal amounts loaded per experiment. Different electron transport pathway states were assessed utilizing protocols designed to measure Complex I- and Complex II-linked activity using subsequent infusions of substrates as listed per figure. Respiration rate was standardized to background oxygen consumption via antimycin A and normalized to mg mitochondrial protein added. Furthermore, a series of experiments were conducted to test the effect of hypoxia/reperfusion on isolated mitochondria by energizing the mitochondria and allowing oxygen in the chamber to deplete. Reoxygenation occurred for 1 min and ROS production was calculated during the reoxygenation phase.

### 2.3. Isolated heart perfusion

Langendorff heart preparations were made from hearts isolated from P20 – P25 *Scn1a*^−/+^ and *Scn1a*^+/+^ mice, similarly to as previously described [20,32]. After a 15-min baseline recording, the perfusate was switched to a buffer containing 80 μM diamide for 30 min. Three blinded reviewers analyzed ECG traces for arrhythmia susceptibility and severity using previously established arrhythmia scoring systems [29].

### 2.4. Myocyte isolation and imaging

P20 – P25 ventricular myocytes were dissociated as previously described [32] and incubated with 500 nM 5-(6)-chloromethyl-2,7-dichlorodihydrofluorescein diacetate (CM-DCF; Invitrogen) [33] before being loaded on a coverslip. Images were acquired on an inverted microscope in 30-s intervals. After 10 min of stable baseline, 80 μM of diamide was added to the recording chamber and images were collected until cell death.

### 2.5. ECG recordings

Surface ECG recordings were performed on lightly anesthetized (1.0 % Isoflurane) mice at P25 using limb leads. At P30-P31 ECG was recorded in unrestrained conscious mice by placing them on the ECGenie Clinic (MouseSpecifics). For both recordings, a stable baseline recording (of at least 5 min) was obtained before injection of 2 mg/kg (i.p) norepinephrine (NE). 10 min after NE injection, 120 mg/kg caffeine (Caff) was injected (i.p.) and recorded for an additional 10 min. Analysis was performed in LabChart (AD Instruments) by three blinded reviewers.

### 2.6. Gene expression via qPCR

RT-qPCR was performed on left ventricular samples from P25 mice using TaqMan gene expression assays (ThermoFisher Scientific). S*od1* (Mm01344232_g1), *Sod2* (Mm01313000_m1), *Gpx* (Mm00656761_g1), and *Gsr* (Mm00439154_m1), with *Tbp* (Mm01277042_m1) as a housekeeping gene.

### 2.7. Statistical analysis

All data are presented as mean ± SEM. A two-way ANOVA (sex × genotype) was first used to determine if sex differences existed. Comparison of respiration and H_2_O_2_ production was performed with a two-way ANOVA (genotype × substrate) with Fisher’s LSD post-hoc. A student’s *t*-test was used for all comparisons between the two groups. Between groups comparison for incidence of whole heart ventricular tachycardia/ventricular fibrillation (ex-vivo) or heart rate fluctuations (in-vivo) was determined using a χ^2^ test. Comparison of cellular survival (Kaplan-Meier curve) was analyzed using a log-rank test. *P* < 0.05 was considered statistically significant.

## 3. Results

### 3.1. Survival of Scn1a^−/+^ mice is decreased following seizure onset

**Fig. 1.**
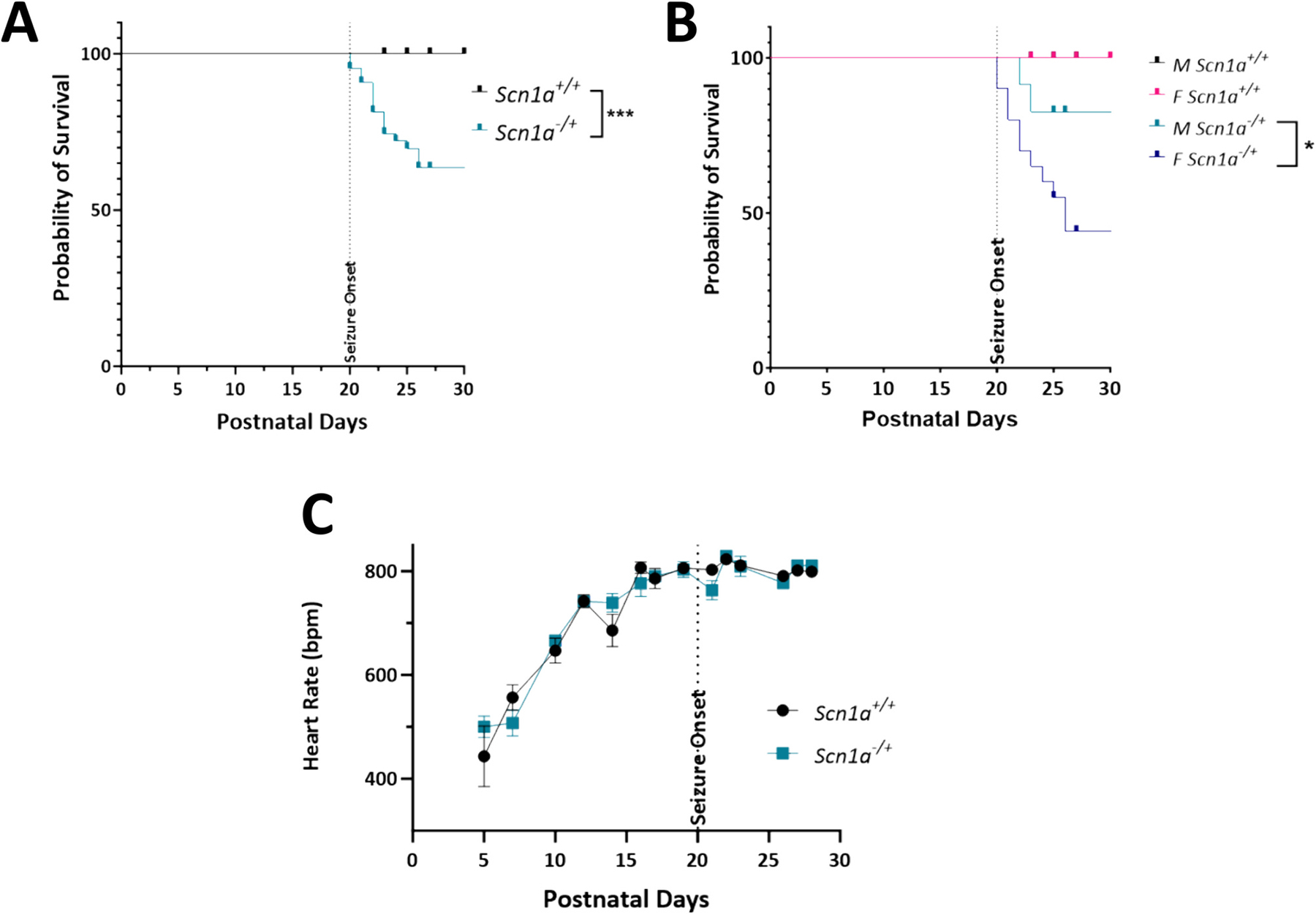
Survival of *Scn1a*^−/+^ mice is decreased compared to *Scn1a*^+/+^ following seizure onset. (A) Survival curve comparing lifespan of *Scn1a*^−/+^ (*N* = 43) and *Scn1a*^+/+^ mice (*N* = 37). Survivability of *Scn1a*^−/+^ is significantly reduced compared to wild-type (*p* = 0.002). (B) In *Scn1a*^−/+^ mice, survivability of female *Scn1a*^−/+^ (*N* = 20) mice is lower than their male (*N* = 23) counterparts (*p* = 0.038). (C) Heart rate of DS mice increases over time, but not significantly compared to *Scn1a*^+/+^ mice. Kaplan-Meier survival curve with Log-rank Mantel-Cox test (A-B) and two-way ANOVA (genotype × time) with Fisher’s LSD post-hoc (C). *, *P* ≤ 0.05.

In the *Scn1a*^−/+^ model, seizures begin approximately 20 days after birth. From this time on, we observed a significant decrease in survival of *Scn1a*^−/+^ mice compared to *Scn1a*^+/+^ (Fig. 1A; *p* = 0.002). This effect was pronounced in the female *Scn1a*^−/+^ cohort, where lifespan was decreased significantly compared to both female *Scn1a*^+/+^ and the male *Scn1a*^−/+^ cohort (Fig. 1B; *p* = 0.04). Importantly, there were no developmental differences in the progression of heart rate increases in *Scn1a*^−/+^ mice (Fig. 1C; *p* = 0.87).

### 3.2. Mitochondrial respiratory chain function is decreased in Scn1a^−/+^ hearts

**Fig. 2.**
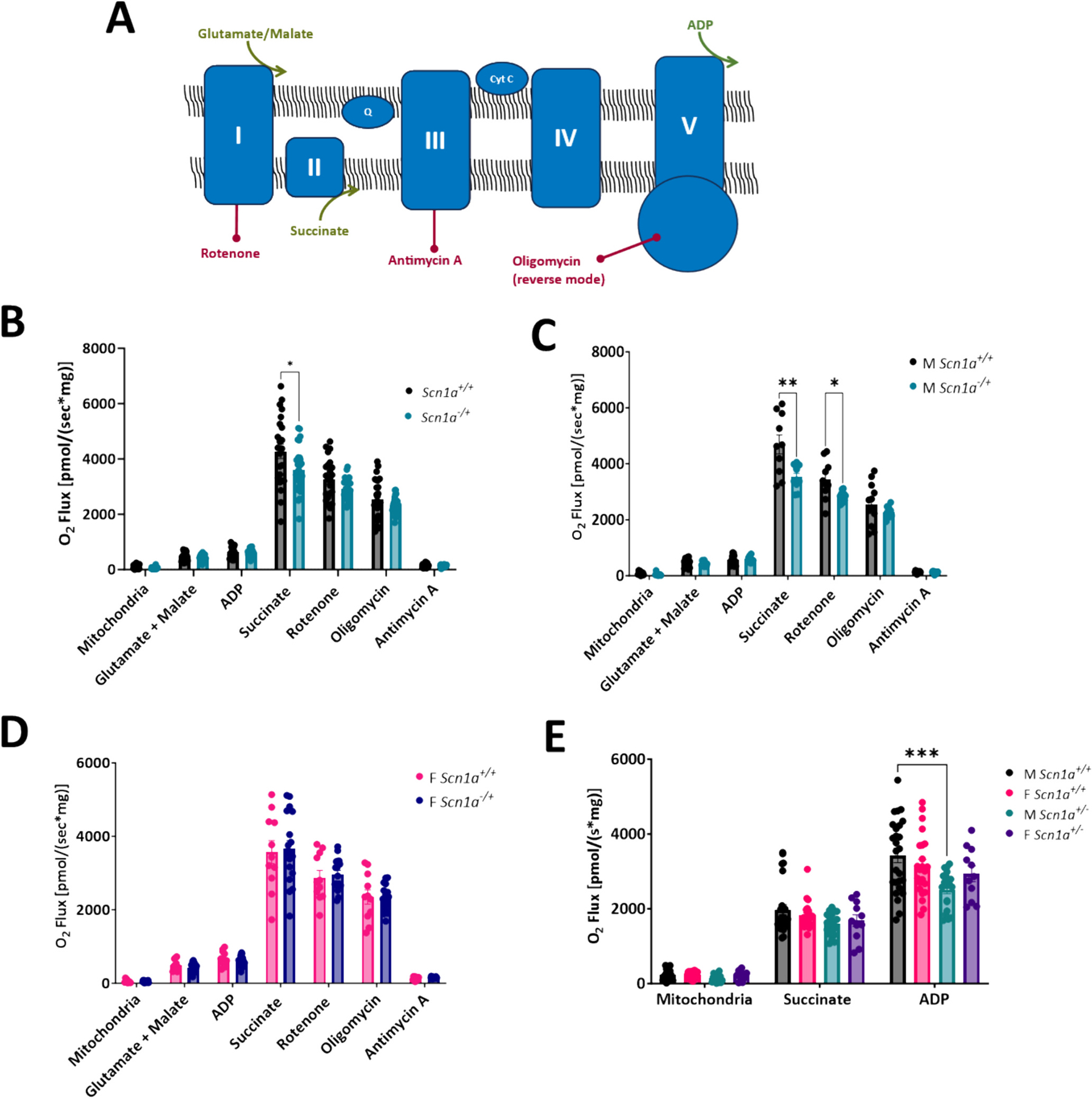
Male *Scn1a*^−/+^ mice have deficits in Complex II-linked respiration. (A) Schematic of oxidative phosphorylation and substrates used in protocols. (B) N- and S-linked substrates were titrated to isolated mitochondria, and oxygen consumption measured using an O2k-FluoRespirometer. No differences in respiratory chain activity were detected between *Scn1a*^−/+^ (*N* = 9; *n* = 29) and *Scn1a*^+/+^ (*N* = 8; *n* = 23) samples (*p* = 0.06–0.48). (C) Deficits in electron transfer states supporting both Complex I- and Complex II-linked respiration (*p* = 0.02) as well as Complex II-linked respiration were observed in mitochondria isolated from male *Scn1a*^−/+^ (N = 4, *n* = 11) hearts compared to male *Scn1a*^+/+^ (N = 4, *n* = 12) hearts (p = 0.03). (D) No differences in Complex I- or Complex II-linked respiration were observed between female *Scn1a*^−/+^ (*N* = 5, *n* = 18) hearts compared to female *Scn1a*^+/+^ (N = 4, n = 11) hearts (*p* = 0.14–0.90). (E) To verify the deficits observed in Complex-II linked respiration in male *Scn1a*^−/+^ mice, S-linked substrates were added to isolated mitochondria in the absence of N-linked substrates. Upon titration of succinate + ADP, oxygen consumption was decreased in mitochondria from male *Scn1a*^−/+^ (*N* = 7, *n* = 26) hearts compared to male *Scn1a*^+/+^ (N = 7, *n* = 20) hearts (*p* = 0.0002). No differences were observed in female *Scn1a*^−/+^ (N = 5, n = 11) hearts compared to female *Scn1a*^+/+^ (N = 9, n = 23) hearts (*p* = 0.44). Two-way ANOVA (genotype × substrate) with Fisher’s LSD post-hoc (B-E). *, P ≤ 0.05, **; *P* ≤ 0.01, ***; *P* ≤ 0.001.

We investigated mitochondrial respiration chain activity under different electron transport states. Respiratory chain substrates and inhibitors were added to parallel chambers of an Oroboros Oxygraph to isolate the activity of Complex I- and Complex II-linked respiration in isolated mitochondria samples (Fig. 2A). First, the NADH-linked substrates glutamate and malate (G/M) were simultaneously added to isolated mitochondria samples to stimulate electron flow into Complex I. Upon the addition of G/M, no differences in oxygen consumption were detected between *Scn1a*^+/+^ and *Scn1a*^−/+^ samples (473.07 ± 30.72 in *Scn1a*^+/+^ vs. 427.27 ± 18.91 in *Scn1a*^−/+^; *p* = 0.19). Next, ADP was added to the energized mitochondria to stimulate oxidative phosphorylation. However, we detected no differences in oxygen consumption between *Scn1a*^+/+^ and *Scn1a*^−/+^ samples in the presence of G/M + ADP (625.94 ± 37.98 in *Scn1a*^+/+^ vs. 594.13 ± 23.95 in *Scn1a*^−/+^; *p* = 0.48), suggesting Complex-I linked ATP production is unchanged between *Scn1a*^+/+^ and *Scn1a*^−/+^ hearts. Therefore, to next assess Complex II-linked respiration, we added saturating levels of succinate, the endogenous substrate of Complex II, to isolated mitochondria. The addition of succinate after G/M and ADP leads to the simultaneous activation of both Complex I and II-linked substrate states, and is an estimate of physiologically relevant maximal respiratory capacity. Upon the titration of succinate, oxygen consumption in mitochondria isolated from *Scn1a*^−/+^ hearts were decreased significantly (4277.65 ± 265.34 in *Scn1a*^+/+^ vs. 3612.90 ± 144.92 in *Scn1a*^−/+^; *p* = 0.03) (Fig. 2B). Lastly, rotenone (an inhibitor of Complex I) was added to our isolated mitochondria samples. After rotenone addition, oxygen consumption was reduced similarly between *Scn1a*^+/+^ and *Scn1a*^−/+^ samples. Oxygen consumption under this state reflects rates of Complex II-linked respiratory activity was not different between *Scn1a*^+/+^ and *Scn1a*^−/+^ (3255.10 ± 161.25 in *Scn1a*^+/+^ vs. 2922.39 ± 66.08 in *Scn1a*^−/+^; *p* = 0.06). Next, oligomycin and antimycin A were added to isolated mitochondrial samples to inhibit reverse ATP synthase activity and measure background O_2_ consumption. No differences in oxygen consumption between DS and wild-type samples were observed after titration of these substrates (*p* values = 0.29–0.70).

### 3.3. Sex differences in mitochondrial respiration in male Scn1a^−/+^ hearts but not female

Initially, as described above, we detected a difference in oxygen consumption when Complex I- and Complex II-linked respiratory pathways were engaged, but no differences were found when these pathways were engaged separately in *Scn1a*^−/+^ cardiac mitochondria. Interestingly, upon the analysis of our respirometry data by sex, we uncovered mitochondria respiratory deficits in male *Scn1a*^−/+^ hearts, but not females. No differences in Complex I-linked ATP production were detected in male *Scn1a*^−/+^ hearts. Upon the addition of N-linked substrates G/M, and ADP, respiration was unaffected in mitochondria from male *Scn1a*^−/+^ hearts compared to *Scn1a*^+/+^ (585.24 ± 48.41 in *Scn1a*^+/+^ vs. 596.18 ± 33.08 in *Scn1a*^−/+^; *p* = 0.86). However, upon the simultaneous engagement of both Complex-I and Complex-II linked electron transfer pathways via the addition of G/M, ADP, and succinate to mitochondria, oxygen consumption in mitochondria from male *Scn1a*^−/+^ hearts were significantly reduced (4692.97 ± 342.12 in *Scn1a*^+/+^ vs. 3534.90 ± 126.11 in *Scn1a*^−/+^; *p* = 0.01). The subsequent addition of rotenone to block Complex I activity revealed that oxygen consumption under the S-pathway control state was significantly lower in mitochondria from male *Scn1a*^−/+^ hearts (3430.72 ± 219.64 in *Scn1a*^+/+^ vs. 2839.87 ± 54.47 in *Scn1a*^−/+^; *p* = 0.03) (Fig. 2C). These results indicated that a deficit in Complex II-linked respiration in our male cohort was likely responsible for this decreased oxygen consumption observed in *Scn1a*^−/+^ cardiac mitochondria during our assessment of mitochondrial respiratory capacity. No differences in mitochondrial respiration were detected between mitochondria isolated from female *Scn1a*^+/+^ and *Scn1a*^−/+^ hearts (*p* = 0.14–0.90) (Fig. 2D). To confirm these sex-specific deficits in *Scn1a*^−/+^ cardiac mitochondria, we analyzed Complex II-linked respiration through an alternative protocol, where succinate + ADP was added directly to isolated mitochondria, in the absence of NADH-linked substrates or rotenone. Upon the titration of these substrates, oxygen consumption was significantly decreased in mitochondria from male *Scn1a*^−/+^ hearts compared to *Scn1a*^+/+^ (3425.93 ± 191.92 in *Scn1a*^+/+^ vs. 2512.06 ± 111.85 in *Scn1a*^−/+^; *p* = 0.02) (Fig. 2E). As in the initial protocol, there were no differences in oxygen consumption in mitochondria from female mouse hearts (3148.82 ± 171.14 in *Scn1a*^+/+^ vs. 2936.78 ± 210.86 in *Scn1a*^−/+;^ *p* = 0.44).

### 3.4. Increased susceptibility to spontaneous arrhythmogenic events under increased sympathetic activation in male Scn1a^−/+^ mice

**Fig. 3.**
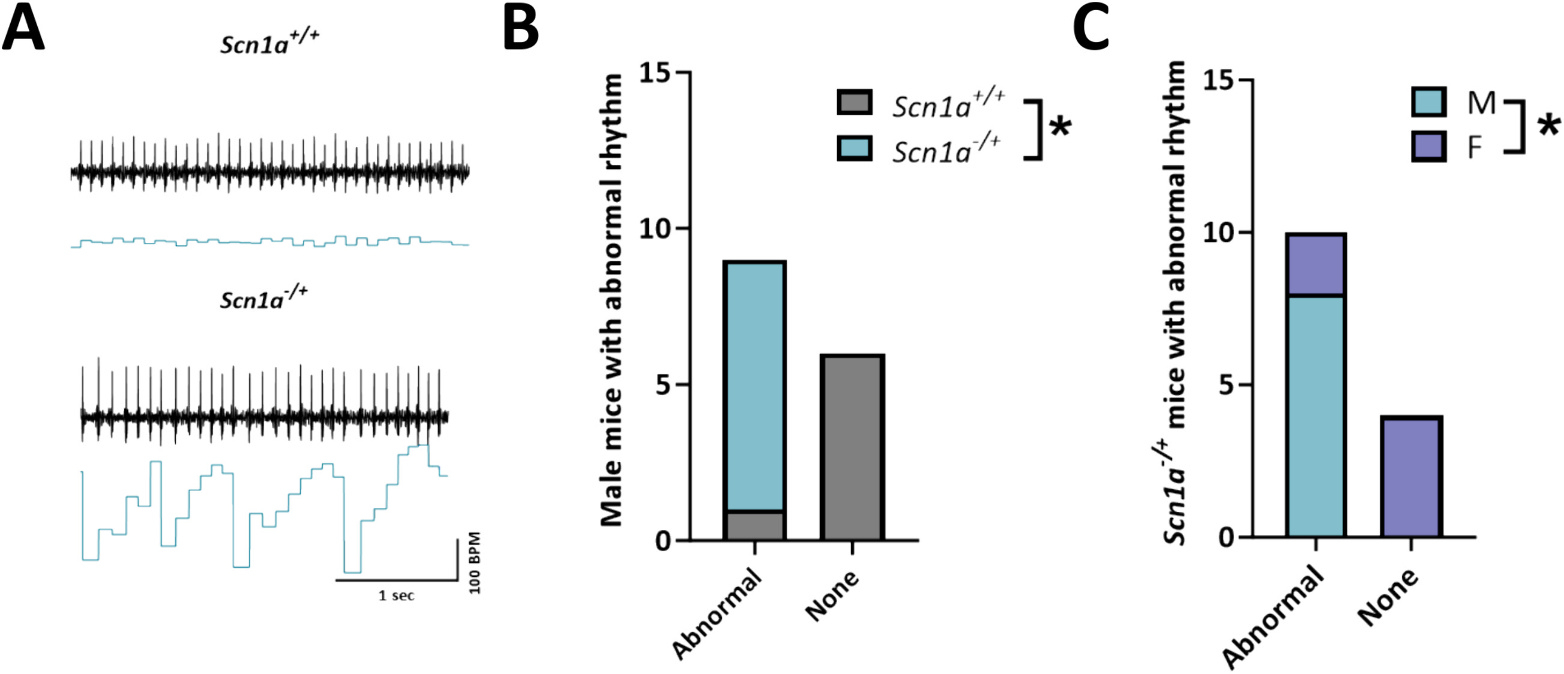
Abnormal cardiac rhythm following norepinephrine (NE) injection in male Scn1a^−/+^ mice. (A) Representative traces from *Scn1a*^+/+^ and *Scn1a*^−/+^ mice. (B) Every male *Scn1a*^−/+^ mouse exhibited abnormal rhythm following NE injection. This observation was significantly more likely than in *Scn1a*^+/+^ male mice. (C) Comparison of male vs female Scn1a^−/+^ mice demonstrate that the abnormal rhythm was significantly more likely to occur in male vs. female mice. Fisher’s exact test (B–C). *, P ≤ 0.05. (N = 7 *Scn1a*^+/+^ male; 8 *Scn1a*^−/−^ male; and 6 *Scn1a*^−/−^ female).

Our previous data indicated contradictory results that mortality in female *Scn1a*^−/+^ mice is increased compared to males, but deficits in Complex II-linked respiration are exclusively in *Scn1a*^−/+^ males. Given that Complex II (succinate dehydrogenase) is responsive to increases in activity during times of increased workload, this observation raised the possibility that mortality in female *Scn1a*^−/+^ may proceed through non-SUDEP epileptic mechanisms (i.e. status epilepticus) whereas in males, death occurs more through cardiac arrhythmias and SUDEP. Therefore, we hypothesized that that under times of increased physiological stress or demand on the heart would reveal an underlying predisposition to cardiac arrhythmias in male but not female *Scn1a*^−/+^ mice. Furthermore, in another epileptic encephalopathy model, we have shown that arrhythmias may develop under conditions that simulate a sympathetic surge [20]. To test this, we subjected lightly anesthetized mice to a 2 mg/kg norepinephrine (NE) followed by 120 mg/kg caffeine (Caff) challenge. Following the injection of NE, we observed an increased probability of spontaneous abnormal rhythm events in male *Scn1a*^−/+^ mice (Fig. 3). Contrary to our observations in the *Scn8a* model [20], differences were primarily seen following NE injection, and we saw no further rhythm disturbances following Caff injection in *Scn1a*^−/+^ mice. Since anesthetized mice have a much slower heart rate than conscious mice, we next assessed heart rhythm under physiological conditions. After allowing mice to recover for 5 days we measured unrestrained conscious ECG recordings. In this setting, we observed the same abnormal rhythmic pattern in male *Scn1a*^−/+^ mice. Male *Scn1a*^−/+^ were much more likely to experience dysrhythmias than female *Scn1a*^−/+^ mice (*p* = 0.01) in conscious mice as well. Taken together these results suggest that abnormal cardiac rhythm is more likely to occur during period of high sympathetic levels across multiple experimental conditions.

### 3.5. Mitochondrial reactive oxygen species production is unchanged between Scn1a^−/+^ and Scn1a^+/+^ hearts, regardless of sex

**Fig. 4.**
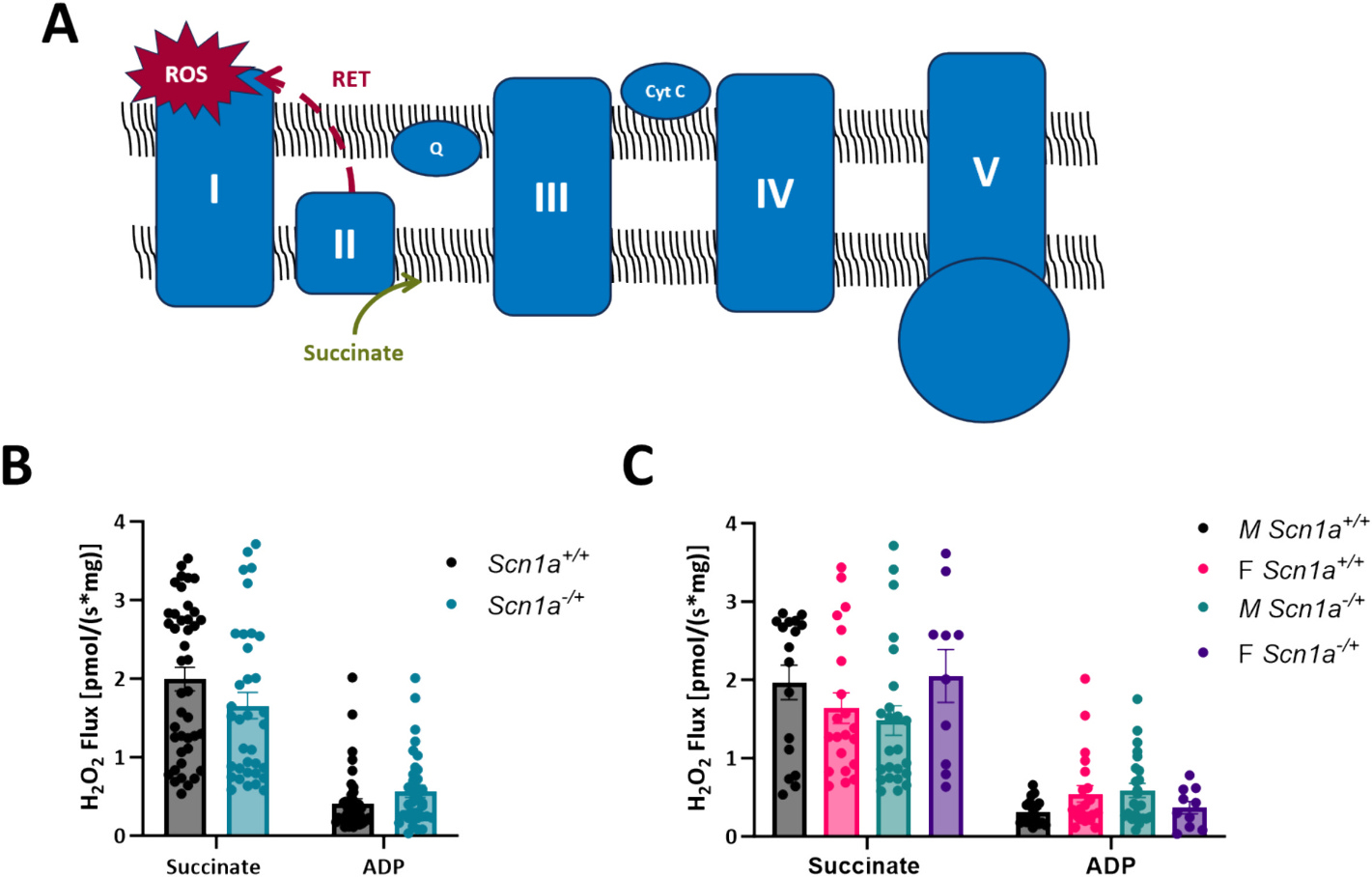
No differences in mitochondrial reactive oxygen species production between *Scn1a*^−/+^ and *Scn1a*^+/+^ mice. (A) Schematic of reverse electron flow. (B) Isolated mitochondria were analyzed for reactive oxygen species production (ROS) under conditions of reverse electron transfer (addition of succinate). Although elevated, no significant differences in ROS production were observed between mitochondria from *Scn1a*^−/+^ (*N* = 12, *n* = 35) and *Scn1a*^+/+^ (*N* = 16, *n* = 42) hearts (*p* = 0.052). (C) When data was separated by sex, there were no differences in ROS production in male *Scn1a*^−/+^ (N = 7, *n* = 24) hearts compared to male *Scn1a*^+/+^ (N = 7, *n* = 17) hearts (*p* = 0.10), or between female *Scn1a*^−/+^ (N = 5, *n* = 10) hearts compared to female *Scn1a*^+/+^ (N = 9, *n* = 21) hearts (p = 0.14). Two-way ANOVA (genotype × substrate) with Fisher’s LSD post-hoc (B–C).

Elevated levels of mitochondrial ROS production can lead to the accumulation of oxidative species and oxidative stress. Therefore, alongside our respirometry experiments, we also investigated if there are differences in ROS production in mitochondria isolated from *Scn1a*^+/+^ and Scn*1a*^−/+^ mouse hearts (Fig. 4). Specifically, we were interested in measuring mitochondrial ROS production under conditions of reverse electron transfer (RET) of electrons to Complex I. We chose to investigate mitochondrial ROS production under this state, as RET is the major pathway for pathological ROS generation within mitochondria. The large amounts of ROS produced during RET have been previously associated with cardiac injury [34,35]. To generate conditions supporting RET, saturating levels of succinate were added to isolated mitochondrial preparations. However, upon succinate titration, ROS production was not different between mitochondria isolated from *Scn1a*^−/+^ and *Scn1a*^+/+^ hearts (1.99 ± 0.15 in *Scn1a*^+/+^ vs. 1.66 ± 0.17 in *Scn1a*^−/+^; *p* = 0.052) (Fig. 4B). Contrary to our respirometry experiments, this observation held even after separating our data by sex, where mitochondrial ROS production between male ROS production was not greater in mitochondria isolated from male *Scn1a*^−/+^ animals compared to samples from *Scn1a*^+/+^ (1.97 ± 0.22 in *Scn1a*^+/+^ vs. 1.48 ± 0.19 in *Scn1a*^−/+^; *p* = 0.10) (Fig. 4C). In female hearts, mitochondria ROS production during RET was unchanged between *Scn1a*^+/+^ and *Scn1a*^−/+^ samples (1.64 ± 0.20 in *Scn1a*^+/+^ vs. 2.05 ± 0.31 in *Scn1a*^−/+^; *p* = 0.14). These results suggest respiratory chain deficits in male *Scn1a*^−/+^ hearts may exist independent of changes in mitochondrial ROS production.

### 3.6. Hypoxia-reoxygenation leads to increased reactive oxygen species production in Scn1a^−/+^ cardiac mitochondria

**Fig. 5.**
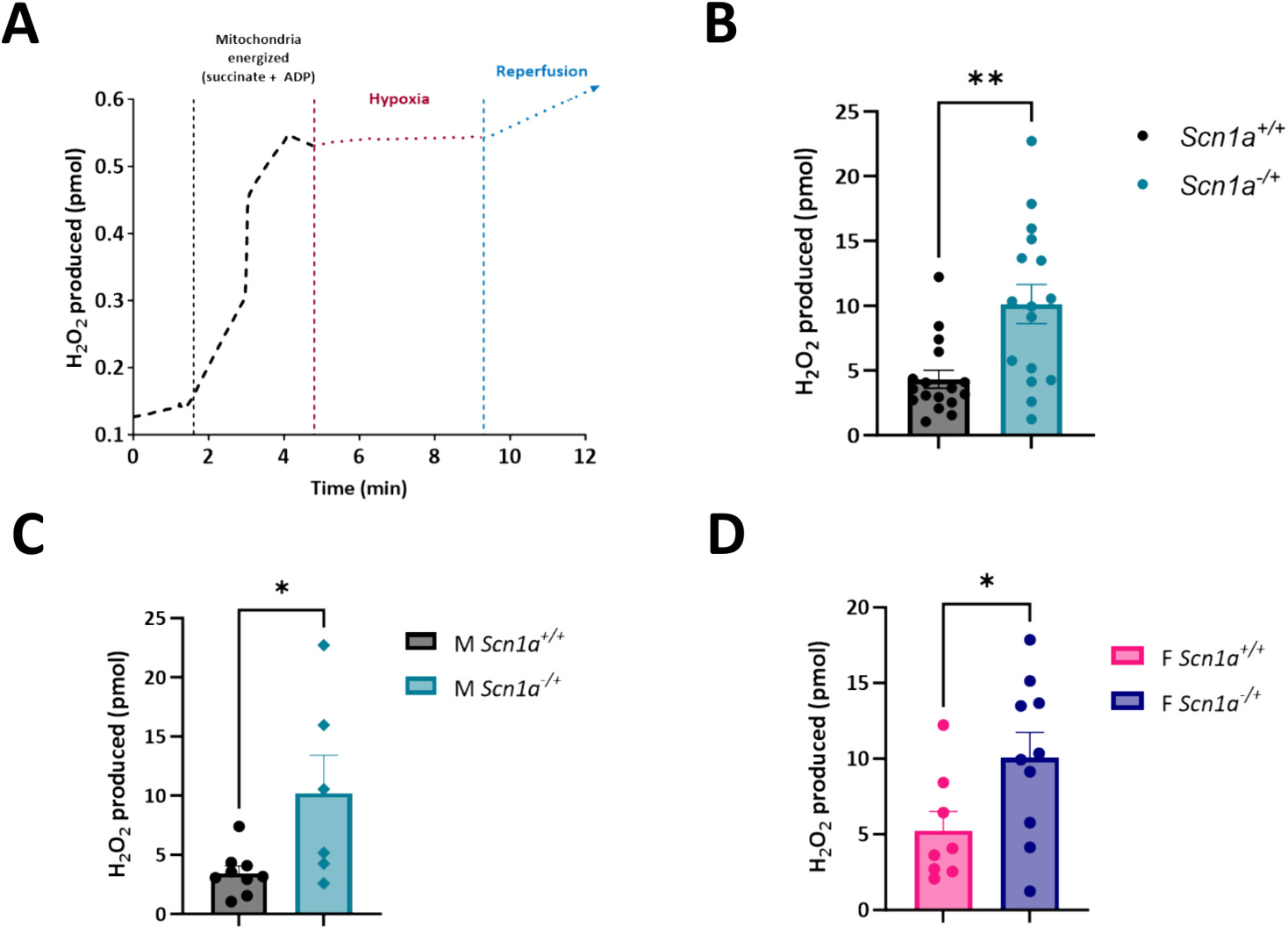
SUDEP in *Scn1a*-linked DS may proceed through a multisystem mechanism. (A) Example trace of a mitochondrial sample undergoing hypoxia/reperfusion. Isolated mitochondria from *Scn1a*^−/+^ and *Scn1a*^+/+^ hearts were energized with succinate + ADP, then subjected to a five-minute period of hypoxia in a O2k-FluoRespirometer, followed by one minute of reperfusion. (B) Mitochondrial reactive oxygen species production during reperfusion was significantly elevated in *Scn1a*^−/+^ (N = 9, *n* = 16) compared to *Scn1a*^+/+^ (N = 8, n = 17) (*p* = 0.001). Upon separating data by sex, ROS production was higher in both mitochondrial samples from male *Scn1a*^−/+^ hearts (N = 4; *n* = 6) (p = 0.03) (C) and female *Scn1a*^−/+^ hearts (N = 5; n = 10) (*p* = 0.04) (D) compared to their respective wild-type counterparts. *t*-test (B–D). *, P ≤ 0.05; **, *P* ≤ 0.01.

Next, as SUDEP is often a multisystem event, we wanted to test the effect of hypoxic conditions on our mitochondria by simulating a low-oxygen environment (Fig. 5). In addition, Dravet patients have been shown to undergo periods of oxygen desaturation either peri-ictally [36] or during sleep [38,41]. These events have been implicated in SUDEP related mortality in DS [36,39,42]. In addition, RNA-seq data from a mouse model of DS shows that hypoxia pathways are elevated [46]. However, if there is an effect of these hypoxic events on the heart has not been explored. Our previous work has shown that during similar events ROS production and collapses in mitochondrial membrane potential can occur during hypoxia/reoxygenation at the cellular level [37]. Isolated mitochondria from *Scn1a*^+/+^ and *Scn1a*^−/+^ hearts were loaded in parallel chambers of the O2k FluoRespirometer. After energizing mitochondria with succinate and ADP, the oxygen in the recording chamber was allowed to run out, and mitochondria preparations were held in hypoxic conditions for five minutes and then reoxygenated (Fig. 5A). Upon stabilization of the signal, the total ROS (H_2_O_2_) produced during reoxygenation was measured over 1 min. Compared to *Scn1a*^+/+^, ROS produced during hypoxia was increased significantly in mitochondria isolated from *Scn1a*^−/+^ hearts (4.32 ± 2.83 in *Scn1a*^+/+^ vs. 10.13 ± 6.07 in *Scn1a*^−/+^; *p* = 0.001) (Fig. 5B). This effect in DS samples was not sex-dependent; ROS production was increased significantly during reperfusion in mitochondria isolated from male *Scn1a*^−/+^ hearts, as well as (3.47 ± 0.61 in *Scn1a*^+/+^ vs. 10.22 ± 3.21 in *Scn1a*^−/+^; *p* = 0.03) female *Scn1a*^−/+^ hearts, ROS (5.27 ± 1.24 in *Scn1a*^+/+^ vs. 10.07 ± 1.65 in *Scn1a*^−/+^; *p* = 0.04) (Fig. 5C-D).

### 3.7. Cardiomyocytes from male Scn1a^−/+^ hearts have increased susceptibility to thiol oxidation

**Fig. 6.**
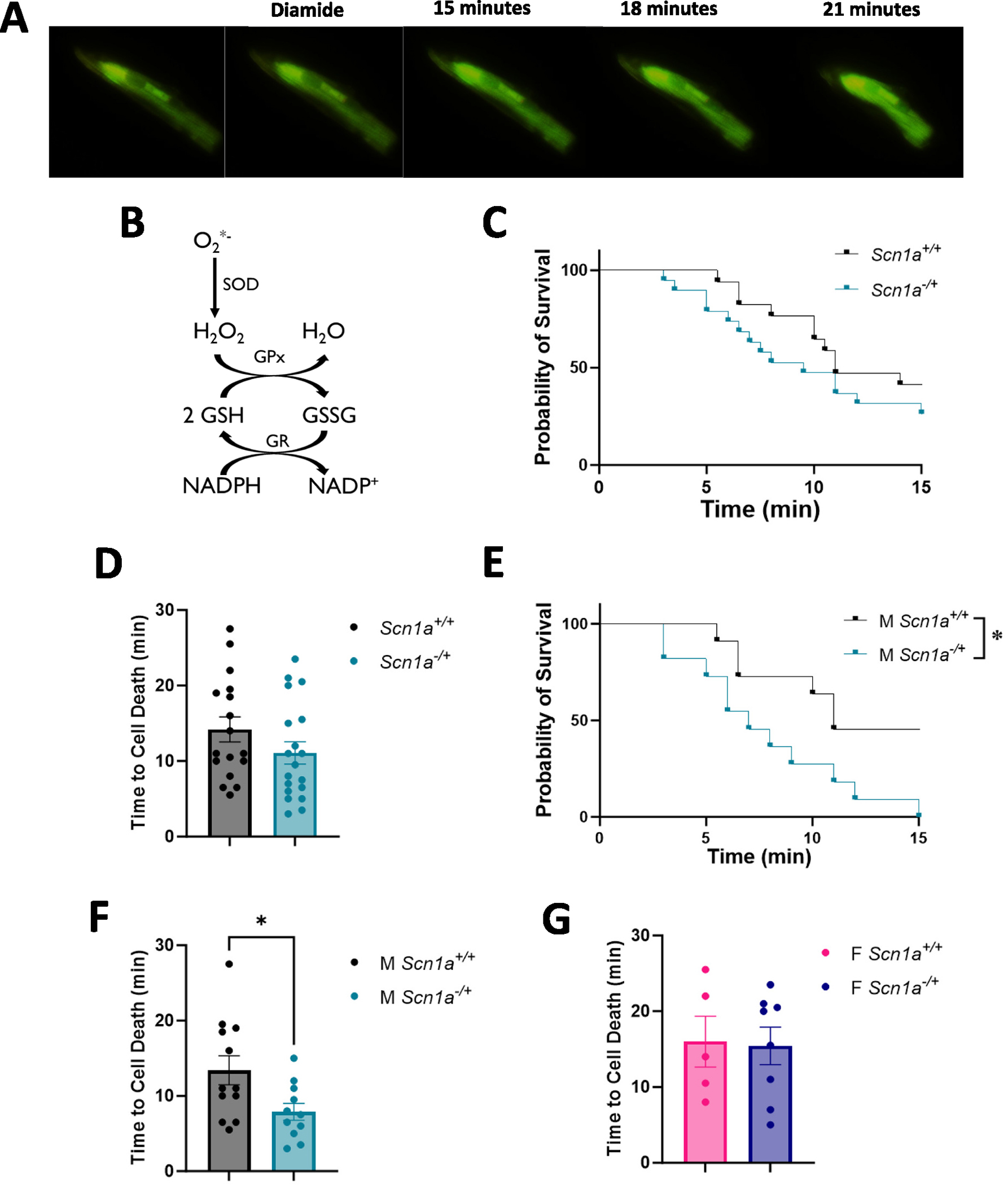
Cardiomyocytes from *Scn1a*^−/+^ mice are more susceptible to thiol oxidation. (A) Isolated primary ventricular cardiomyocytes from *Scn1a*^−/+^ and *Scn1a*^+/+^ hearts were given an 80 սM treatment of the thiol oxidant, glutathione, and analyzed using fluorescent microscopy and the ROS indicator, CM-DCF. (B) The glutathione (GSH) system is the primary thiol buffer in the heart that scavenges ROS and is linked to mitochondrial function. (C) Survival curve comparing lifespan of *Scn1a*^−/+^ (N = 8; *n* = 19) and *Scn1a*^+/+^ (*N* = 10; n = 17) cardiomyocytes following diamide treatment. Survivability of *Scn1a*^−/+^ cells is not significantly reduced compared to *Scn1a*^+/+^ (*p* = 0.20). (D) The average time to cell death between *Scn1a*^−/+^ and *Scn1a*^+/+^ cardiomyocytes is not significantly different (*p* = 0.17). (E) Survival of cells from male *Scn1a*^−/+^ (N = 5; n = 11) mice is significantly lower than those from male *Scn1a*^+/+^ (N = 5; n = 12) mice following diamide treatment, along with average time to cell death (p = 0.03) (F). (G) Average time to cell death was similar between cardiomyocytes isolated from female *Scn1a*^−/+^ (N = 3; *n* = 8) mice compared to cells isolated from wild-type (N = 5; *n* = 5) (*p* = 0.89). Kaplan-Meier Survival curve with Log-rank Mantel-Cox test (C, E), t-test (D, F, G). *, P ≤ 0.05.

After our determination that there were no differences in mitochondrial ROS production under basal conditions, we next wanted to establish if there are differences in cellular ROS scavenging and/or accumulation in *Scn1a*^−/+^ cardiomyocytes under stress (Fig. 6). Similar to our previous work [33], we specifically tested the proficiency of the glutathione (GSH) redox buffer within cardiomyocytes. The GSH system is closely linked to mitochondrial function [40,43], and is one of the main antioxidant pathways within the heart [44]. We isolated primary ventricular cardiomyocytes from *Scn1a*^+/+^ and *Scn1a*^−/+^ hearts and loaded cells with CM-DCF, a fluorescent indicator of global ROS within cells. Fluorescent microscopy was used to assess changes in the DCF signal and detect cell death (Fig. 6A). After an initial recording period to ensure cell stability, we treated cardiomyocytes with 80uM of diamide. Diamide slowly oxidizes thiols, like GSH, and therefore presents a prolonged oxidative challenge to the cell and a test of the strength of its GSH system (Fig. 6B). There was no difference in survivability between DS and wild-type cells (*p* = 0.20) (Fig. 6C). In cardiomyocytes from *Scn1a*^−/+^ mice, we found that, while decreased, the average time to cell death was not significantly lower compared to those from *Scn1a*^+/+^ hearts (14.18 ± 1.65 in *Scn1a*^+/+^ vs. 11.08 ± 1.47 in *Scn1a*^−/+^; *p* = 0.17) (Fig. 6D). However, upon the separation of data by sex, we once again detected deficits in samples from male *Scn1a*^−/+^ mice (*p* = 0.01) (Fig. 6E). Cardiomyocytes isolated from male *Scn1a*^−/+^ hearts died on average 5.82 min sooner than cells isolated from *Scn1a*^+/+^ following diamide treatment (13.42 ± 1.93 in *Scn1a*^+/+^ vs. 7.91 ± 1.12 in *Scn1a*^−/+^; *p* = 0.02) (Fig. 6F). These results indicate that male *Scn1a*^−/+^ animals may possess a weakened GSH system or an increased susceptibility to thiol oxidation, and therefore a reduced ability to handle ROS accumulations and oxidative stress. No significant difference in cell survivability was observed between cardiomyocytes isolated from female *Scn1a*^−/+^ mice compared to *Scn1a*^+/+^ (16.00 ± 3.35 in *Scn1a*^+/+^ vs. 15.44 ± 2.47 in *Scn1a*^−/+^; *p* = 0.89) (Fig. 6G).

### 3.8. Expression of antioxidant genes is increased in male Scn1a^−/+^ hearts

**Fig. 7.**
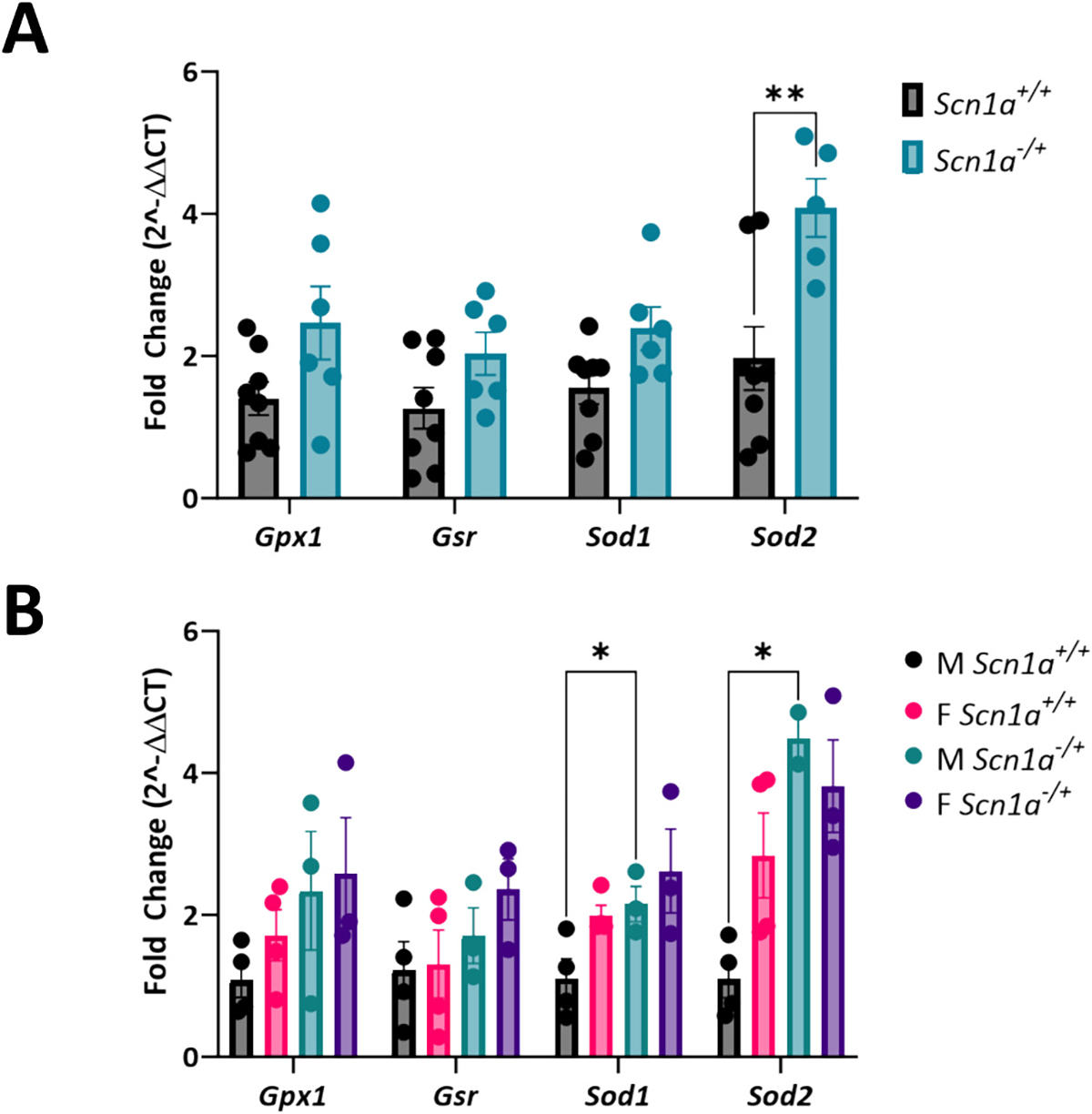
Increased expression of antioxidant genes in male *Scn1a*^−/+^ hearts. (A) The expression of antioxidant genes *Gpx*, *Gsr*, *Sod1*, and *Sod2* was evaluated in left ventricular samples from *Scn1a*^−/+^ (*N* = 6) and *Scn1a*^+/+^ (N = 8) hearts. Expression of *Sod1* was significantly elevated in *Scn1a*^−/+^ samples (*p* = 0.005). (B) Evaluation of gene expression by sex revealed that the expression of *Sod1* (p = 0.04) and *Sod2* (*p* = 0.01) is decreased in male *Scn1a*^−/+^ hearts (N = 3) compared to *Scn1a*^+/+^ (N = 4). Two-way ANOVA (genotype × gene) (A) or (genotype + sex × gene) (B) with Fisher’s LSD post-hoc. *, P ≤ 0.05; **, P ≤ 0.01.

Following our findings that male *Scn1a*^−/+^ cardiomyocytes have reduced capacity to handle thiol oxidation, we next wanted to determine any underlying differences in the expression of antioxidant enzymes (Fig. 7). We used RT-qPCR to quantify the expression of GSH system antioxidant enzymes, glutathione reductase (*Gsr*), and glutathione peroxidase (*Gpx*) as well as the expression of the cytosolic and mitochondrial isoforms of superoxide dismutase, *Sod1* and *Sod2*. Upon comparing gene expression in *Scn1a*^−/+^ and *Scn1a*^+/+^ hearts, we found that expression of *Sod2* was significantly increased in *Scn1a*^−/+^ samples (2.08-fold increase in *Scn1a*^−/+^; *p* = 0.005) (Fig. 7A). Upon separating our data by sex, we found that male *Scn1a*^−/+^ hearts were the main driver of this difference (4.09-fold increase in male *Scn1a*^−/+^; *p* = 0.01) (Fig. 7B). These analyses also showed that *Sod1* expression is increased in male *Scn1a*^−/+^ hearts (4.09-fold increase in male *Scn1a*^−/+^; *p* = 0.04). No differences in the expression of genes were observed between female *Scn1a*^−/+^ and *Scn1a*^+/+^ hearts (*p* = 0.16–0.40).

### 3.9. Susceptibility to arrhythmias under thiol oxidation is unchanged in hearts from Scn1a^−/+^ mice compared to Scn1a^+/+^

**Fig. 8.**
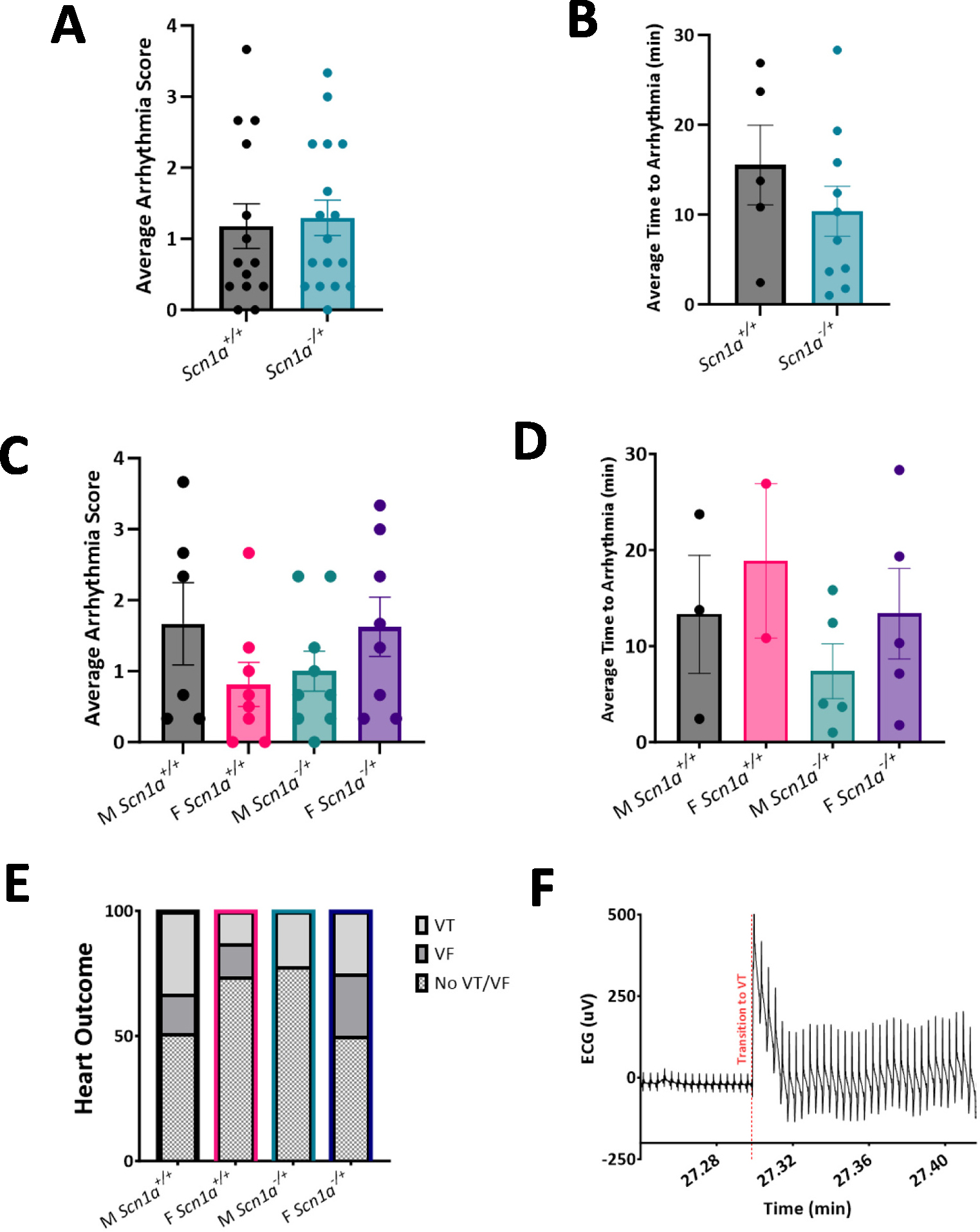
No differences in susceptibility to thiol oxidation were observed in whole hearts. (A) ECG traces collected from isolated hearts perfused with diamide for 30 min were scored for arrhythmia incidence. No difference in this score was observed between *Scn1a*^−/+^ (*N* = 17) and *Scn1a*^+/+^ (*N* = 14) hearts (*p* = 0.77). (B) Average time to arrhythmia incidence after the onset of diamide perfusion is unchanged between *Scn1a*^−/+^ and *Scn1a*^+/+^ hearts (*p* = 0.70). (C) No sex-dependent differences in arrhythmia scores were detected between male *Scn1a*^−/+^ (N = 9) and *Scn1a*^+/+^ (N = 6) hearts (*p* = 0.27), or female *Scn1a*^−/+^ (N = 8) and *Scn1a*^+/+^ (N = 8) hearts (p = 0.14). (D) No sex-dependent differences in time to arrhythmia onset were detected between male *Scn1a*^−/+^ and *Scn1a*^+/+^ hearts (*p* = 0.40), or female *Scn1a*^−/+^ and *Scn1a*^+/+^ hearts (*p* = 0.50). (E) Incidence of potentially fatal arrhythmias, ventricular tachycardia (VT) or ventricular fibrillation (VF), between *Scn1a*^−/+^ and *Scn1a*^+/+^ cohorts. (F) ECG trace from a *Scn1a*^−/+^ heart showing an episode of VT. t-test (A-B), one-way ANOVA with Fisher’s LSD post-hoc (C–D), and Fisher’s exact test (E).

From the results of our cellular data, we hypothesized that the increased cell death in male *Scn1a*^−/+^ myocytes following diamide treatment may translate to an increased susceptibility to arrhythmia in whole hearts (Fig. 8). To test this theory, we excised hearts from DS mice and retrograde perfused hearts with 80 mM diamide for 30 min while collecting continuous ECG measurements. The resulting ECG signal was then scored similar to our previous work [45]. Our results indicated were no significant differences in arrhythmia occurrence between *Scn1a*^−/+^ and *Scn1a*^+/+^ mice following diamide treatment (average arrhythmia score 1.179 ± 0.31 in *Scn1a*^+/+^ vs. 1.29 ± 0.25 in *Scn1a*^−/+^; *p* = 0.77) (Fig. 8A). There was also a similar incidence of arrhythmia between sample groups. Of hearts that did experience VT or VF, the time between diamide administration and the first incidence of arrhythmia was also not significantly shorter in *Scn1a*^−/+^ mice (15.53 ± 4.43 in *Scn1a*^+/+^ vs. 10.38 ± 2.78 in *Scn1a*^−/+^; *p* = 0.70) (Fig. 8B). Once data was separated by sex, it appeared hearts from *Scn1a*^−/+^ male mice are not more susceptible to arrhythmia following oxidative stress than those from *Scn1a*^+/+^. Arrhythmia scores (average arrhythmia score 1.67 ± 0.58 in *Scn1a*^+/+^ vs. 1.00 ± 0.28 in *Scn1a*^−/+^; *p* = 0.25) (Fig. 8C) as well as average time to arrhythmia following diamide perfusion (Fig. 8D) was unchanged in *Scn1a*^−/+^ hearts. Similarly, average arrhythmia score was not different between female *Scn1a*^−/+^ and *Scn1a*^+/+^ hearts (0.81 ± 0.31 in *Scn1a*^+/+^ vs. 1.63 ± 0.42 in *Scn1a*^−/+^; *p* = 0.14). Contrary to our predictions, the female *Scn1a*^−/+^ mice were the main driver of arrhythmias in the DS cohort. 50 % of *Scn1a*^−/+^ female hearts experienced an episode of VT or VF during the recording period, compared to only 22 % of male *Scn1a*^−/+^ hearts (Fig. 8E). These observations are contrary to our mitochondria data, which suggested a more extreme phenotype in male *Scn1a*^−/+^ mice. These results indicate there may be distinct cardiac mechanisms leading to arrhythmia and SUDEP between male and female *Scn1a*^−/+^ animals.

## 4. Discussion

In this study, we sought to determine if alterations in mitochondrial bioenergetics and reactive oxygen species are present in a mouse model of *Scn1a*-linked DS, as these pathways may contribute to the development of arrhythmias and SUDEP. Our main findings demonstrate that male *Scn1a*^−/+^ Dravet mice are more susceptible to deficits in mitochondrial ATP production, ROS balance, and cardiac arrhythmias. Our male mice showed a deficit in their ability to generate ATP through Complex II-mediated respiration, without a change in ROS production. We then showed that *Scn1a*^−/+^ mice have a decreased ability to buffer ROS accumulation, alongside altered expression of antioxidant enzymes. Lastly, spontaneous arrhythmogenic events were significantly increased under conditions of physiological stress in male, but not female *Scn1a*^−/+^ mice. Despite an earlier mortality rate in female mice, our data demonstrates that male *Scn1a*^−/+^ mice are more susceptible to cardiac arrhythmias. Given the complex nature of SUDEP and mortality in DS, we have shown that sex differences may play an important role in risk factors associated with early mortality in DS. This work further supports evidence that multiple physiological mechanisms may contribute to how cardiac arrhythmias have the potential to underly SUDEP in DS.

We initially detected a difference in respiratory capacity between *Scn1a*^−/+^ and *Scn1a*^+/+^ cardiac mitochondria when Complex I- and Complex II-linked electron transfer pathways were simultaneously engaged. Upon analyzing data by sex, we found this effect was driven by respiratory deficits in Complex II-linked respiration, specifically in the male *Scn1a*^−/+^ cohort. Overall, a decreased energy supply within the heart can be dangerous. Upon β-adrenergic stimulation of the heart, the cAMP-dependent protein kinase A (PKA) is activated. PKA phosphorylates multiple Ca^2+^ handling proteins within the excitation-contracting (EC) coupling process, which increases cellular Ca^2+^ flux. Elevated Ca^2+^ concentrations within the cell leads to increased sequestration of Ca^2+^ via the mitochondrial Ca^2+^ uniporter, the primary mitochondrial Ca^2+^ uptake pathway. Ca^2+^ then stimulates the activity of citric acid cycle enzymes, and consequently, electron transfer through Complex II [47,48]. Therefore, deficits in Complex II-linked respiration may lead to a mismatch between cardiac demand and output. Our data support this observation, as we showed male *Scn1a*^−/+^ mice develop abnormal rhythms under β-adrenergic stimulation with a decreased ability to increase heart rate. Deficits in this pathway could be especially detrimental to patients with DS, especially during epileptic episodes, which can activate autonomic control centers in the brain.

SUDEP is often believed to result from the culmination of postictal depression of neuronal reflex centers, along with cardiac and respiratory arrest [6]. The heart depends on oxidative phosphorylation as it possesses only a small ATP reserve and is thus exceptionally vulnerable to hypoxic conditions. In a cohort of patients with genetic epilepsies, hypercapnia occurring during seizures was found to lower the threshold for cardiac arrhythmias [49]. Therefore, in this study, we decided to perform experiments testing the effect of hypoxia on isolated cardiac mitochondria. Our results indicated that mitochondria from *Scn1a*^−/+^ hearts have a reduced ability to handle hypoxia/reperfusion injury than their wild-type counterparts, regardless of sex. The increased ROS production by *Scn1a*^−/+^ during hypoxia/reperfusion may highlight the importance of the multisystem nature of SUDEP in generating adverse cardiac events. Especially, as we observed no differences in ROS production during RET in mitochondria from *Scn1a*^−/+^ at steady normoxic conditions.

In many of our experiments, we observed sex differences in mitochondrial and cellular responses, specifically in our male *Scn1a*^−/+^ cohort. In male *Scn1a*^−/+^ hearts, respiration is compromised through Complex II-linked pathways. Furthermore, male cardiomyocytes had increased sensitivity to thiol oxidation, alongside elevated activation of antioxidant genes, suggesting an increased baseline of oxidative stress. The effect of sex chromosomes on epilepsy and SUDEP is not extensively studied. However, biological sex may be an important consideration in the development of new therapeutics to prevent seizures and SUDEP. Differences in response to antiepileptics have been found to occur due to patient sex [50]. In DS, one group has reported that there may be a slightly higher incidence of *Scn1a*-linked DS in males [51], although majorly, DS is believed to impact both sexes equally [52–54]. Furthermore, it has been reported that SUDEP susceptibility in *Scn1a*-linked DS is roughly equal among the sexes [55]. Contrarily, in the *Scn1a*^−/+^ mouse model, female mice were found to experience more severe seizures resulting in a greater SUDEP risk [56]. This latter observation is consistent with our data, where female *Scn1a*^−/+^ exhibited decreased survival following the onset of seizures, even when compared to male *Scn1a*^−/+^ animals. The increased death rate in female *Scn1a*^−/+^ mice may be due to an increased number of status epilepticus events, as opposed to a SUDEP mechanism. This is an important distinction for our findings, as the cause of death across our mice was not determined to be SUDEP (via cardiac arrhythmias or other causes), increased seizure burden, or other cause of death. However, our data suggest that male *Scn1a*^−/+^ mice may be more susceptible to cardiac arrhythmias following onset of seizures. These sex differences may have important implications for an increased need to examine cardiac abnormalities and treatments in the clinical population, especially in the males.

Biological sex may also exert structural and physiological changes in the brain that impact epilepsy etiology [57]. Human male and female genomes produce sex-specific signals that influence gene activation. Many of these sex-specific signals play significant roles in development and early life and therefore may be especially pertinent in juvenile epilepsies such as DS. Androgens, like testosterone, have been linked to increased seizure susceptibility due to their neuroexcitatory effects [58–60]. Sex hormones are also known to influence neuronal activity by affecting gene expression and chromatin packaging, modulating membrane excitability through binding membrane receptors [61,62]. These effects can also be temporal, as X- and Y-linked factors are differentially expressed among brain structures [52]. Diverging epileptic phenotypes between sexes may also impact the heart. The hippocampus is a structure often linked to epileptic networks due to its recurrent circuits and is also part of the limbic system of higher autonomic control of the heart. An increased number of cells in the hippocampus are generated during the perinatal period in male animals, eventually developing into mature neurons at juvenility [63,64]. In our study we often saw exacerbated effects in males; however, further research is needed to clarify the conflicting data on sex on SUDEP risk in *Scn1a*-linked DS.
